# Point-of-Care Ultrasound-Guided Hydrodissection in Emergency Care: A Structured Narrative Review of Perineural and Related Musculoskeletal Applications

**DOI:** 10.7759/cureus.114161

**Published:** 2026-08-08

**Authors:** Carlos A Gonzalez-Cobos, Juan Cruz Lopez, Francisco A Gonzalez, Gabriel Rose, Miguel F Agrait Gonzalez

**Affiliations:** 1 Emergency Medicine, Centro Medico Episcopal San Lucas, Ponce, PRI; 2 Emergency Medicine, Ponce Health Sciences University, Ponce, PRI; 3 Emergency Medicine, Kaiser Permanente, San Diego, USA

**Keywords:** emergency medicine, fascial plane hydrodissection, hydrodissection, musculoskeletal ultrasound, perineural hydrodissection, peripheral nerve entrapment, peripheral nerve hydrodissection, point-of-care-ultrasound, ultrasound guided nerve block

## Abstract

Background: Point-of-care ultrasound (POCUS)-guided hydrodissection is the intentional injection of fluid under real-time ultrasound guidance to separate a peripheral nerve or another defined tissue plane from adjacent structures. The role of hydrodissection in emergency care remains uncertain.

Methods: We conducted a structured narrative review. PubMed/Medical Literature Analysis and Retrieval System Online (MEDLINE) was searched from database inception through July 23, 2026, using a reproducible query covering ultrasound-guided hydrodissection of peripheral nerves and muscular, fascial, tendon-sheath, and peritendinous targets. Citation tracking supplemented the database search. Two authors performed the primary screening, and three additional authors evaluated the included records; disagreements were resolved by discussion with final adjudication by the lead author. Of 138 PubMed records and seven records identified through citation tracking, 32 publications were retained for narrative synthesis. Acute-care reports were appraised with design-appropriate Joanna Briggs Institute (JBI) tools.

Results: Eight acute-care hydrodissection reports involving 23 patients were identified: four perineural reports, three non-perineural muscular or fascial reports, and one mixed nerve-adjacent/fascial report. One additional emergency department (ED) sciatic nerve-block series was retained only as related procedural context because intentional tissue-plane separation was not documented. The acute-care evidence comprised four case reports, three case series, and one retrospective observational cohort. Reports described short-term pain or functional improvement, but the evidence was limited by uncontrolled designs, heterogeneous targets and injectates, brief or incomplete follow-up, confounding co-interventions, selective reporting, and incomplete adverse-event ascertainment. JBI appraisal identified generally adequate descriptions of patients and procedures but recurrent uncertainty regarding consecutive or complete inclusion, standardized case identification, and adverse-event assessment.

Conclusions: Small uncontrolled reports suggest that POCUS-guided hydrodissection is technically feasible in selected acute-care presentations and may be followed by short-term symptom improvement. They do not establish efficacy, comparative benefit, durability, or safety. Perineural, non-perineural, and related non-hydrodissection procedures should be interpreted separately. At present, POCUS-guided hydrodissection should be considered an investigational procedural approach rather than routine emergency care.

## Introduction and background

Point-of-care ultrasound (POCUS) has become an integral component of contemporary emergency care, supporting rapid bedside assessment, procedural guidance, and clinical decision-making across a broad range of time- sensitive presentations. Contemporary clinical studies continue to evaluate the diagnostic reliability and operational performance of POCUS, including a recent randomized trial comparing three POCUS techniques for confirmation of endotracheal tube placement [1]. Ultrasound-guided regional anesthesia has also expanded options for procedural anesthesia and multimodal, opioid-sparing analgesia in the emergency department (ED) [2].

Hydrodissection is a related but distinct procedural concept. It involves intentional fluid-mediated separation of a nerve or another defined tissue plane under real-time ultrasound visualization. In perineural hydrodissection, the target is the plane surrounding a peripheral nerve. Proposed mechanisms include mechanical separation from adjacent fascia or scar, reduction of focal compression, restoration of nerve mobility, improved local microvascular flow, and injectate-specific effects. These mechanisms remain incompletely established and likely vary by target, diagnosis, chronicity, and injectate [3,4].

Physiologic rationale and safety considerations

Perineural hydrodissection is intended to create or restore an extraneural tissue plane, not to distend the nerve or force injectate into an intraneural space. Under continuous ultrasound visualization, fluid is injected incrementally adjacent to the epineurium, with visible separation of the nerve from surrounding fascia, tendon, or scar tissue serving as the intended procedural endpoint. Sonographic nerve expansion, severe pain or paresthesia, unexpected resistance, or failure of the tissue plane to separate should prompt immediate cessation rather than increasingly forceful injection. This technical distinction does not eliminate the possibility of a transient local pressure increase, but it differentiates controlled extraneural tissue-plane separation from intraneural injection, which carries a substantially different risk profile [3,4].

The proposed mechanical rationale is that reducing focal tethering or adhesion may restore nerve mobility and relieve compression of the nervi nervorum and vasa nervorum, thereby improving intraneural microcirculation and reducing ischemia-related nociceptive signaling [3,4]. In a cadaveric study of 12 wrists, ultrasound-guided hydrodissection reduced peak median nerve gliding resistance by 21.4% compared with no meaningful change in untreated controls [5]. This finding supports mechanical plausibility but does not establish clinical benefit or safety because the specimens were unaffected by carpal tunnel syndrome, injection pressures were not measured, and neural function could not be evaluated.

The concern that injected fluid may increase pressure around an already compromised nerve remains physiologically relevant. Experimental studies demonstrate that the magnitude and duration of compression influence neural dysfunction [6] and that high-pressure intraneural injection can produce fascicular injury [7]. However, the clinical hydrodissection studies identified in this review did not measure compartment or injection pressures, establish pressure-time safety thresholds, or incorporate continuous electrophysiologic monitoring. These remain important evidence gaps rather than evidence that the procedure is either physiologically harmless or inherently injurious. The 2024 American Academy of Orthopaedic Surgeons guideline concludes that hydrodissection does not provide established long-term benefit for carpal tunnel syndrome, while its supporting rationale acknowledges individual studies demonstrating improved patient-reported outcomes at three to six months [8]. Separately, a systematic review of 10 clinical studies reported favorable short-term outcomes and no serious adverse events, but its limited sample sizes, heterogeneous techniques, and incomplete adverse-event surveillance preclude a definitive conclusion regarding safety [9]. Accordingly, outpatient and experimental evidence provides a rationale for continued investigation but cannot establish safety, efficacy, or routine applicability in emergency care.

Most clinical evidence comes from outpatient pain medicine, sports medicine, physical medicine and rehabilitation, and musculoskeletal ultrasound practice. Several systematic reviews have focused principally on carpal tunnel syndrome or peripheral entrapment neuropathies [9-12]. Those reviews describe heterogeneous techniques and generally low-to-moderate certainty evidence, with the most developed comparative literature involving outpatient carpal tunnel syndrome. They do not establish effectiveness in acute-care populations.

The present review adds three elements not emphasized in prior syntheses: a specific focus on procedures performed during ED or urgent acute-care encounters; explicit separation of perineural hydrodissection, non-perineural hydrodissection, and related ultrasound-guided injections that do not meet a hydrodissection definition; and discussion of implementation, evidence limitations, and research priorities for emergency clinicians. Our objective was to describe, classify, and critically appraise the available acute-care literature without extrapolating outpatient efficacy or safety claims to ED practice.

## Review

Materials and methods

Review Design and Search Strategy

We conducted a structured narrative review rather than a systematic review or meta-analysis. PubMed/Medical Literature Analysis and Retrieval System Online (MEDLINE) was searched from database inception through July 23, 2026. Removing the previous January 2010 lower date limit avoided an arbitrary temporal exclusion. The complete PubMed query was: ("hydrodissection"[Title/Abstract] OR "hydro-dissection"[Title/Abstract] OR hydrodissect*[Title/Abstract]) AND (ultrasound[Title/Abstract] OR ultrasonograph*[Title/Abstract] OR sonograph*[Title/Abstract] OR "point-of-care ultrasound"[Title/Abstract] OR POCUS[Title/Abstract]) AND (nerve[Title/Abstract] OR perineural[Title/Abstract] OR neuralgia[Title/Abstract] OR neuropath*[Title/Abstract] OR entrapment[Title/Abstract] OR "carpal tunnel"[Title/Abstract] OR sciatica[Title/Abstract] OR radicul*[Title/Abstract] OR tendon*[Title/Abstract] OR tenosynov*[Title/Abstract] OR fascial[Title/Abstract] OR fascia[Title/Abstract] OR myofascial[Title/Abstract] OR muscular[Title/Abstract] OR muscle[Title/Abstract] OR piriformis[Title/Abstract] OR musculoskeletal[Title/Abstract]) AND English[Language] AND humans[MeSH Terms].

Additional records were evaluated using the same eligibility and classification criteria. PubMed was the only bibliographic database searched; therefore, the review should not be interpreted as a comprehensive systematic identification of all potentially eligible evidence.

Eligibility, Screening, and Data Extraction

Eligible publications were peer-reviewed English-language adult human clinical reports addressing therapeutic ultrasound-guided hydrodissection of a peripheral nerve or a muscular, fascial, tendon-sheath, or peritendinous plane. Systematic reviews and selected comparative outpatient studies were retained to establish context. Conference abstracts, preprints, pediatric-only studies, nontherapeutic cadaveric studies, tumor-ablation or surgical hydrodissection, and regional anesthesia studies without an intended therapeutic hydrodissection endpoint were excluded.

Juan Cruz Lopez and Francisco A. Gonzalez performed the primary title/abstract and full-text review. Records retained after primary review were evaluated by Carlos A. Gonzalez-Cobos, Miguel F. Agrait Gonzalez, and Gabriel Rose. Discrepancies were resolved through discussion, with final adjudication by Dr. Gonzalez-Cobos as lead author. The same process was applied to records identified through citation tracking. Extracted variables included study design, sample size, actual clinical setting, operator background, target and procedural classification, injectate, local anesthetic and corticosteroid use, sonographic endpoint, outcomes, adverse-event ascertainment, follow-up, and principal limitations.

**Figure 1:**
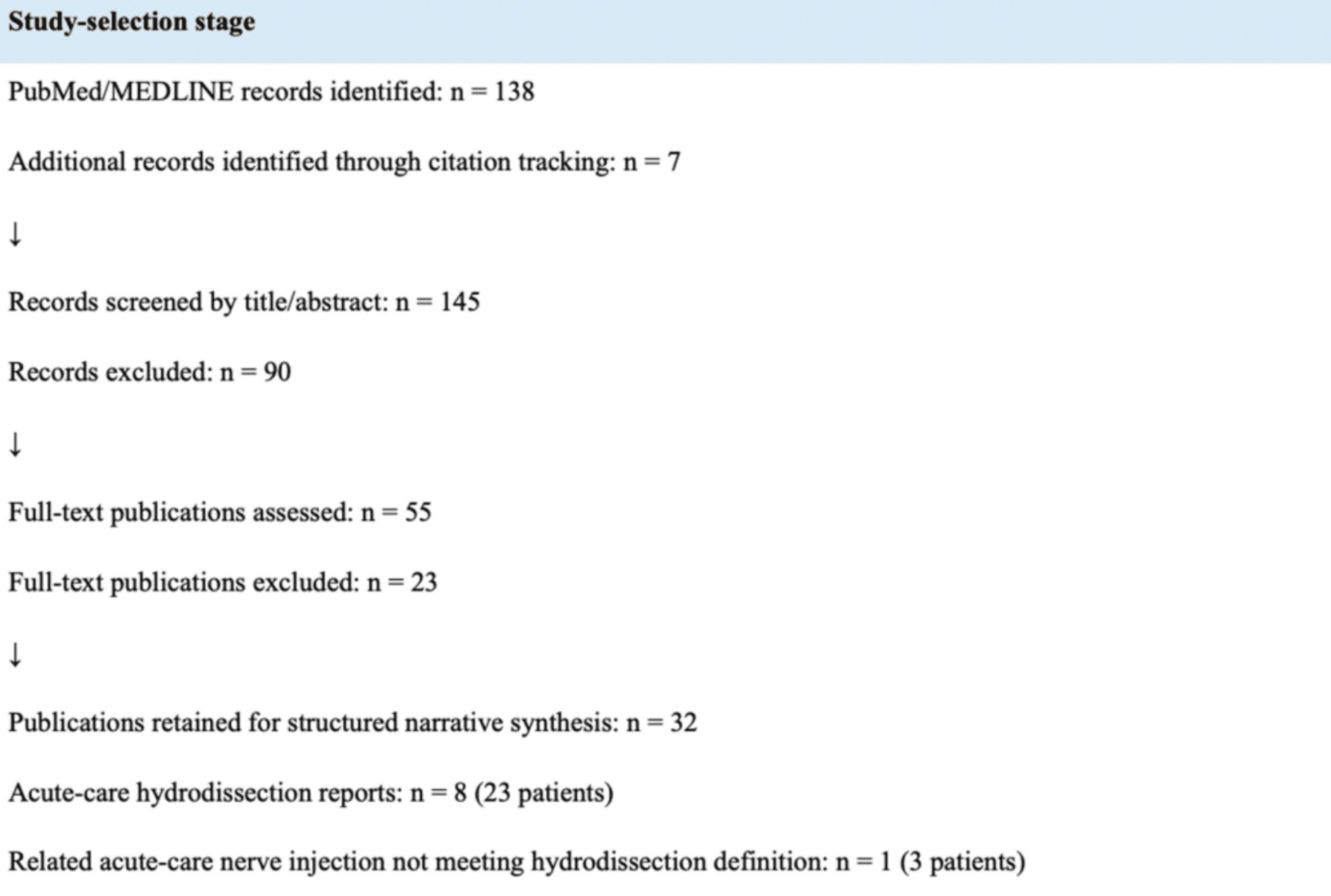
Study selection flow diagram Counts reflect the updated search conducted till July 23, 2026. MEDLINE: Medical Literature Analysis and Retrieval System Online

The search retrieved 138 PubMed records; seven additional records were identified through citation tracking. Ninety records were excluded at title/abstract review. Fifty-five full texts were assessed, 23 were excluded after full-text review, and 32 publications were retained for the structured narrative synthesis. Eight were acute-care hydrodissection reports. One additional ED nerve-block series was retained as related procedural context but was not counted as hydrodissection (Figure 1).

Procedural and Setting Classification

Hydrodissection required source-level evidence of intentional fluid-mediated separation of the target from adjacent tissue or a documented sonographic separation endpoint. Procedures were assigned to one of three categories: Perineural hydrodissection: intentional fluid separation around an identified peripheral nerve. Non-perineural hydrodissection: intentional separation of a muscular, fascial, tendon-sheath, or peritendinous plane. Related ultrasound-guided nerve or fascial injection not clearly meeting the definition of hydrodissection: an ultrasound-guided block or nerve-adjacent injection without documented intentional tissue-plane separation.

Clinical setting, rather than operator specialty, determined acute-care classification. A publication was classified as emergency or acute care only when the procedure occurred during an ED or urgent acute-care encounter. Operator specialty and ultrasound experience were recorded separately.

Methodological Appraisal and Synthesis

Acute-care single-patient reports were appraised with the Joanna Briggs Institute (JBI) Checklist for Case Reports, and uncontrolled multi-patient reports were appraised with the JBI Checklist for Case Series [13,14]. We did not calculate aggregate quality scores because item-level judgments are more informative than a summed score. The complete appraisal appears in Appendix A.

Clinical and methodological heterogeneity precluded statistical pooling. We, therefore, used structured narrative synthesis organized by procedural category and clinical setting.

Results

Evidence Outside Emergency Care

The most developed comparative literature concerns outpatient carpal tunnel syndrome. Systematic reviews and network meta-analyses have evaluated saline, corticosteroid, 5% dextrose in water (D5W), platelet-rich plasma, and other injectates, but the contributing trials differ in technique, comparator, volume, follow-up, and outcome measurement [9-12]. Some trials report improvement after D5W perineural injection or hydrodissection compared with control or corticosteroid injection [15,16]. These findings provide biologic and procedural rationale for further study, but they do not establish that D5W is superior in acute-care populations.

Injectate selection in emergency care remains unresolved. Saline may provide mechanical separation without a pharmacologic analgesic effect. Local anesthetics can provide immediate analgesia but confound interpretation and introduce dose-related toxicity. Corticosteroids may contribute delayed benefit and their own adverse effects. D5W has been investigated extensively in outpatient carpal tunnel syndrome, but no ED comparative evidence establishes its superiority over saline, local anesthetic, or other injectates.

Non-perineural applications include interfascial treatment for myofascial pain and tendon-sheath or peritendinous procedures. Controlled and observational outpatient studies have reported interfascial hydrodissection for shoulder, neck, and lumbodorsal myofascial pain and hydrodissection for de Quervain tenosynovitis [17-19]. These interventions differ from perineural hydrodissection in target, mechanism, comparator, and outcome. They support the broader procedural concept of ultrasound-guided tissue-plane separation but should not be used as direct evidence for perineural efficacy or acute-care safety.

Acute-Care Evidence and Procedural Classification

Eight acute-care hydrodissection reports involving 23 patients met the review definition (Table 1) [20-29]. Four were perineural hydrodissection reports (seven patients), three were non-perineural muscular or fascial hydrodissection reports (14 patients), and one was a mixed nerve-adjacent/fascial report (two patients). The evidence comprised four case reports, three case series, and one retrospective observational cohort.

**Table 1:** Acute-care reports classified by procedural definition 5W: 5% dextrose in water; ED: emergency department; JBI: Joanna Briggs Institute; LA: local anesthetic; MRC: Medical Research Council; NRS: Numeric Rating Scale; POCUS: point-of-care ultrasound; h: hours. *At least one author of the cited report is an author of the present review. Eligibility and classification were based on prespecified clinical-setting and procedural criteria, not author identity. These reports describe distinct clinical encounters and were not a coordinated study dataset.

Study, design, and setting	Classification, target, and injectate	Observed outcome, follow-up, and adverse-event assessment	Principal limitations and JBI signal
Aulia et al., 2026 [25]; retrospective observational cohort, n=11; single ED; emergency physicians	Non-perineural paravertebral fascial-plane injection plus fascial needling. Bupivacaine concentration/dilution varied; saline and dexamethasone variably used (LA: yes; steroid: variable).	Median NRS 9 to 3 at discharge and 1 at 24–48 h among 10 followed. Clinical documentation and 48-h health-system return were checked; no complications reported.	Retrospective, uncontrolled, small, and heterogeneous intervention; one patient lost to follow-up; consecutive and complete inclusion not fully clear.
Silver et al., 2023 [20]; case series, n=4; ED; POCUS-trained faculty/fellow	Perineural sciatic nerve hydrodissection with D5W 20 mL (LA: no; steroid: no).	Immediate NRS improvement in all; telephone follow-up at 24–72 h; one recurrence near 72 h. No predefined adverse-event surveillance described.	Selection and completeness unclear; subjective outcomes; no comparator; brief follow-up.
Rose, 2024 [21]; case report, n=1; ED; emergency physician	Perineural greater occipital nerve hydrodissection with documented muscle-layer separation; 9 mL saline + 1 mL 1% lidocaine (LA: yes; steroid: no).	Pain 9/10 to 1/10 at 10 min; relief at 72 h. No structured adverse-event surveillance described.	Single case; anesthetic confounding; short follow-up; published correspondence identified image-labeling concerns.
Fuchs and Rose, 2024 [22]; case report, n=1; ED; emergency physicians	Perineural median nerve hydrodissection for carpal tunnel syndrome with D5W 8 mL (LA: no; steroid: no).	Pain 8/10 to 2/10 at 15 min with improved paresthesia; relief at 72 h. No structured adverse-event surveillance described.	Single case; subjective outcome; short follow-up; durability unknown.
Fuchs et al., 2024 [24]; case series, n=2; ED; emergency physicians	Mixed nerve-adjacent/fascial hydrodissection of spinal accessory and dorsal scapular nerve regions; 5 mL 0.5% bupivacaine + 15 mL D5W divided between planes (LA: yes; steroid: no).	Immediate pain improvement; relief at 48–72 h. No predefined adverse-event surveillance described.	Mixed targets; anesthetic confounding; selection/completeness unclear; no comparator.
Kaga, 2022 [26]; case report, n=1; initial ED encounter with subsequent outpatient procedures; emergency/general internal medicine physician	Non-perineural fascial hydrodissection of the semispinalis capitis–obliquus capitis inferior plane and deep sternocleidomastoid; initial 0.75% ropivacaine, later 9 mL saline + 1 mL 1% lidocaine (LA: yes; steroid: no).	Immediate improvement; repeat procedures; no recurrence at four weeks after analgesic discontinuation. No adverse events explicitly reported.	Single case; repeated interventions; medication co-intervention; short follow-up.
Kaga and Ueda, 2022 [27]; case series, n=2; Department of Emergency and General Internal Medicine; one admission	Non-perineural hydrodissection of superficial/deep piriformis surfaces with 18 mL saline + 2 mL 1% lidocaine (LA: yes; steroid: no).	Immediate NRS improvement; one repeat injection; four-week follow-up in one patient and incomplete follow-up after discharge in the other. No structured adverse-event surveillance described.	Two patients; repeat procedure and physiotherapy; incomplete follow-up; published figure correction.
Marrero Borrero et al., 2026 [23]; case report, n=1; ED; emergency physician	Perineural radial nerve hydrodissection with documented tissue separation; 8 mL D5W + 2 mL 1% lidocaine + 1 mL dexamethasone 4 mg/mL (LA: yes; steroid: yes).	Partial improvement at 24 h; MRC 3/5 at six weeks; reported resolution at three months. No predefined adverse-event surveillance described.	Single case; natural neurapraxia recovery; aspiration, splinting, and physical therapy confound attribution.
Goldsmith et al., 2020 [29]; case series, n=3; ED; emergency physicians	Related procedure, not hydrodissection: transgluteal sciatic nerve block without documented separation; 15 mL 0.5% bupivacaine + dexamethasone 10 mg (LA: yes; steroid: yes).	Immediate pain reduction and improvement at 24 h. No predefined adverse-event surveillance described.	Conventional nerve block, not direct hydrodissection evidence; high local-anesthetic exposure; short follow-up.

The perineural reports addressed severe acute sciatica, post-traumatic occipital neuralgia, carpal tunnel syndrome, and delayed radial neuropathy [20-23]. All described intentional separation or a sonographic tissue-plane endpoint. Immediate improvement was reported, but follow-up ranged from 24 hours to three months and co-interventions included local anesthetic, corticosteroid, splinting, aspiration, and physical therapy. Natural recovery was a particularly important alternative explanation in the delayed radial neuropathy report.

The mixed nerve-adjacent/fascial report described combined spinal accessory and dorsal scapular nerve-region hydrodissection for refractory upper back pain [24]. Because both neural and fascial targets were treated and local anesthetic was used, the clinical response cannot be attributed to perineural hydrodissection alone.

The non-perineural reports included paravertebral interfascial injection with fascial needling, fascial hydrodissection for occipital neuralgia, and hydrodissection of the superficial and deep piriformis muscle surfaces [25-27]. In the occipital case, the initial procedure occurred in the ED, but repeated procedures occurred in an outpatient general internal medicine setting. The targeted structures were the plane between the semispinalis capitis and obliquus capitis inferior muscles and the deep surface of the sternocleidomastoid; these were therefore classified as fascial rather than perineural hydrodissection. The piriformis report was classified as muscular/fascial hydrodissection rather than sciatic nerve hydrodissection. Its published erratum corrected reversed medial/lateral and ischium/sacrum labels in the figures [28].

Related ED literature has described ultrasound-guided transgluteal sciatic nerve analgesia for refractory back pain [29]. The report documented a perineural sciatic nerve injection with bupivacaine and dexamethasone but did not clearly document intentional tissue-plane separation. It is therefore included only as related procedural context and not as direct evidence for hydrodissection. Similarly, an outpatient report of acute radial nerve palsy demonstrated intentional perineural hydrodissection but was not classified as acute care because the clinical setting was not an ED or urgent acute-care encounter (Table 1) [30].

Methodological Appraisal

The JBI case-report appraisal found that demographics, clinical history, current condition, diagnostic assessment, intervention, and post-intervention status were generally described. Adverse-event reporting was less consistent: one report explicitly stated that no adverse events occurred, whereas the other reports did not describe a predefined surveillance method. A published letter concerning the ED occipital report identified image-labeling issues; this did not negate the described fluid-mediated separation endpoint but reduced confidence in anatomical reporting.

Across the case series, the most recurrent appraisal limitations were unclear eligibility criteria, uncertainty about consecutive and complete inclusion, small samples, subjective outcome assessment, and incomplete or brief follow-up. The retrospective Aulia cohort used explicit clinical eligibility and standardized NRS outcomes but remained uncontrolled, used heterogeneous injectates and techniques, and lacked prospective adverse-event ascertainment. These methodological limitations mean the reports can support descriptions of feasibility and observed short-term outcomes only.

POCUS Technique and Injectate Considerations

Before needle placement, the operator should identify the target and surrounding vessels, fascia, tendons, pleura, bone, and other structures relevant to the region. Color Doppler may help identify vessels. Continuous needle-tip visualization and incremental injection are general procedural safety principles. For perineural hydrodissection, the expected sonographic endpoint is visible spread that separates the nerve from adjacent tissue; circumferential or near-circumferential spread may occur, but the required endpoint and volume have not been validated for ED practice.

Needle placement within the nerve should be avoided. Injection should stop if there is high resistance, unexpected severe pain, paresthesia, nerve expansion, or loss of needle-tip visualization. When local anesthetic is used, concentration, total dose, weight-based maximum, incremental administration, and readiness to recognize and treat local anesthetic systemic toxicity must be considered [2,31]. The reviewed literature does not establish a preferred ED injectate or volume.

Proposed Clinical and Implementation Considerations

Hydrodissection should not substitute for evaluation of time-sensitive diagnoses. Based on general principles of ultrasound-guided regional and musculoskeletal procedures, hydrodissection would generally be inappropriate when compartment syndrome, cauda equina syndrome, local infection, unstable traumatic injury, clinically significant bleeding risk, or a neurologic deficit requiring urgent specialist assessment is suspected [2,31]. These are expert-informed safety considerations, not contraindications validated in hydrodissection trials (Table 2).

**Table 2:** Proposed clinical considerations for future evaluation of POCUS-guided hydrodissection in emergency care. This framework is author-proposed, derived from general procedural and emergency diagnostic principles, and has not been prospectively validated. It should not be interpreted as a clinical practice guideline. POCUS: point-of-care ultrasound

Clinical scenario and potential research target	Alternative or time-sensitive diagnoses to evaluate first	Post-procedure and follow-up measures for future study
Focal neuropathic pain or suspected entrapment; identified peripheral nerve	Fracture/dislocation, vascular injury, compartment syndrome, compressive mass, progressive deficit	Repeat pain and neurologic examination; function; splinting or specialty follow-up when indicated
Sciatica-like pain refractory to initial therapy; sciatic nerve or adjacent tissue plane	Cauda equina syndrome, epidural infection, malignancy, fracture, progressive motor deficit	Neurologic reassessment; recurrence; falls or weakness; spine/primary-care follow-up
Occipital neuralgia pattern; greater/lesser occipital nerve or defined fascial plane	Intracranial injury, cervical vascular pathology, meningitis, unstable cervical injury	Headache recurrence, neurologic examination, medication use, repeat-procedure requirement
Upper-extremity weakness or paresthesia; median, radial, ulnar, or other nerve	Fracture/dislocation, cervical radiculopathy or myelopathy, compartment syndrome, structural lesion	Serial motor/sensory examination; function; electrodiagnostic or specialty follow-up
Focal tendon-sheath or peritendinous pain; tendon sheath or peritendinous plane	Septic tenosynovitis, cellulitis/abscess, fracture, tendon rupture	Pain, function, recurrence, infection surveillance, hand/sports-medicine follow-up
Myofascial or fascial-plane pain; defined muscular or fascial interface	Infection, trauma, radiculopathy, cardiopulmonary or visceral source as applicable	Functional reassessment, recurrence, rescue analgesia, physical therapy or primary-care follow-up

Departments evaluating hydrodissection should treat it as an advanced ultrasound-guided procedure. Relevant competencies include regional sonoanatomy, sterile technique, continuous needle visualization, recognition of intraneural or intravascular injection, injectate pharmacology, and post-procedure neurologic and functional reassessment. Simulation, phantom or cadaver training, supervised cases, and image review may be useful, but no validated training threshold, minimum case number, or credentialing standard currently exists for emergency-care hydrodissection.

Discussion

The available acute-care literature is small and clinically heterogeneous. The eight hydrodissection reports cover different nerves, muscular and fascial planes, diagnoses, injectates, volumes, sonographic endpoints, operators, outcome measures, and follow-up periods. The interventions therefore cannot be treated as a single homogeneous therapy. An immediate pain response after a D5W-only procedure is not equivalent to improvement after a mixed anesthetic-steroid injection, and neither is equivalent to functional recovery over weeks in a patient also receiving splinting and rehabilitation.

The literature is highly vulnerable to publication and selective-reporting bias. Technically successful or symptomatically favorable cases are more likely to be submitted and published than failed procedures, transient responses, recurrence, or complications. Adverse-event ascertainment was usually passive or unspecified. The absence of reported complications in very small samples is not evidence of safety.

Confounding also limits causal interpretation. Local anesthetic can explain immediate analgesia; corticosteroid can contribute delayed benefit; systemic analgesia, aspiration, splinting, physical therapy, and natural symptom fluctuation may influence subsequent outcomes. In compressive neurapraxia, spontaneous recovery is a major alternative explanation. Placebo and contextual procedural effects cannot be separated from hydrodissection in uncontrolled reports.

The updated search restored directly relevant ED publications that had been removed during an earlier editorial effort to limit self-citation. Five acute-care reports have author overlap with the present review, but they involve distinct patient encounters and different author groups rather than a coordinated dataset. Excluding them solely because of authorship would omit most directly relevant perineural ED evidence and substitute less relevant outpatient or non-hydrodissection procedures. We therefore retained them under the same setting, procedural definition, and appraisal criteria applied to every report and disclosed the overlap in Table 1. Their inclusion does not increase the strength of inference; all remain low-level, uncontrolled evidence.

Limitations of This Review

This was a structured narrative review, not a systematic review. Only PubMed/MEDLINE was searched, so eligible records indexed in Excerpta Medica database (Embase), Scopus, Web of Science, or other databases may have been missed. Citation tracking partly mitigated but did not eliminate this limitation. English-language and peer-reviewed full-text restrictions may introduce language and publication-status bias. Study selection involved multi-author review but was not conducted within a prospectively registered protocol.

Formal JBI appraisal improved transparency but cannot correct the intrinsic limitations of case reports, small case series, and an uncontrolled retrospective cohort. Appraisal itself involves judgment, and aggregate scores were intentionally avoided. The evidence base was too heterogeneous for statistical pooling. Finally, the classification of mixed nerve-adjacent/fascial techniques required interpretation of procedural descriptions; we used an explicit separation endpoint to minimize misclassification.

Future Research Priorities

Prospective multicenter registries should first define how frequently hydrodissection is attempted, in whom, by operators with what training, and with what immediate and delayed outcomes. Reports should distinguish perineural from non-perineural procedures and record the target, clinical setting, diagnostic criteria, sonographic findings, needle approach, injectate and volume, local anesthetic and steroid exposure, objective separation endpoint, co-interventions, adverse-event surveillance, and follow-up completeness.

Subsequent comparative studies should focus on a single acute-care presentation and compare hydrodissection plus standard care with condition-specific standard care, an ultrasound-guided nerve block, or a credible control procedure. Outcomes should include pain, motor and sensory function, rescue analgesia, ED length of stay, recurrence, return visits, return to work or activity, patient-reported recovery, delayed diagnoses, and predefined adverse events. Follow-up beyond 72 hours is essential.

## Conclusions

Eight small acute-care reports describe POCUS-guided hydrodissection in 23 selected patients across perineural, mixed nerve-adjacent/fascial, and non-perineural applications. These reports demonstrate that intentional ultrasound-guided tissue-plane separation has been performed in acute-care settings and describe short-term symptom or functional improvement. They do not establish efficacy, comparative benefit, durability, or safety.

Perineural hydrodissection, non-perineural muscular or fascial hydrodissection, and conventional ultrasound-guided nerve injections should be classified and interpreted separately. At present, POCUS-guided hydrodissection should be considered an investigational procedural approach rather than routine emergency care.

## Appendices

Appendix A

Complete Search Strategy

Database: PubMed/MEDLINE Coverage: Database inception through July 23, 2026 Date searched: July 23, 2026 Language restriction: English Publication status: Peer-reviewed full-text publications; conference abstracts and preprints were excluded. Population: Adult humans.

Exact PubMed query

("hydrodissection"[Title/Abstract] OR "hydro-dissection"[Title/Abstract] OR hydrodissect*[Title/Abstract]) AND (ultrasound[Title/Abstract] OR ultrasonograph*[Title/Abstract] OR sonograph*[Title/Abstract] OR "point-of-care ultrasound"[Title/Abstract] OR POCUS[Title/Abstract]) AND (nerve[Title/Abstract] OR perineural[Title/Abstract] OR neuralgia[Title/Abstract] OR neuropath*[Title/Abstract] OR entrapment[Title/Abstract] OR "carpal tunnel"[Title/Abstract] OR sciatica[Title/Abstract] OR radicul*[Title/Abstract] OR tendon*[Title/Abstract] OR tenosynov*[Title/Abstract] OR fascial[Title/Abstract] OR fascia[Title/Abstract] OR myofascial[Title/Abstract] OR muscular[Title/Abstract] OR muscle[Title/Abstract] OR piriformis[Title/Abstract] OR musculoskeletal[Title/Abstract]) AND English[Language] AND humans[MeSH Terms]

The terms after the third AND were intentionally expanded beyond perineural procedures to capture muscular, fascial, tendon-sheath, peritendinous, and myofascial applications. No lower publication-date limit was imposed. Reference lists of included acute-care reports and principal reviews were reviewed, and forward citation tracking was performed.

**Table 3:** Selection accounting

Stage	Number
PubMed/MEDLINE records retrieved	138
Additional records identified through backward/forward citation tracking	7
Records screened by title/abstract	145
Records excluded at title/abstract stage	90
Full-text publications assessed	55
Full-text publications excluded	23
Publications retained for structured narrative synthesis	32
Acute-care hydrodissection reports	8
Patients represented in acute-care hydrodissection reports	23
Related acute-care nerve-injection report not meeting hydrodissection definition	1

Principal full-text exclusion categories were: surgical, tumor-ablation, or anesthesia applications outside the review scope; no therapeutic hydrodissection endpoint; cadaveric or technical report without patient outcome; pediatric-only population; or insufficient original clinical data. A publication could meet more than one exclusion criterion; one primary reason was assigned for accounting.
